# Comment on “Biomechanical Evaluation of Preoperative Rehabilitation in Patients of Anterior Cruciate Ligament Injury”

**DOI:** 10.1111/os.12715

**Published:** 2020-06-15

**Authors:** Mingjin Zhong, Kan Ouyang, Wei Lu

**Affiliations:** ^1^ Department of Sports Medicine The First Affiliated Hospital of Shenzhen University, Health Science Center; Shenzhen Second People's Hospital Shenzhen China

Dear Editor,

In a recent issue of *Orthopaedic Surgery*, Li W and his colleagues published an article entitled “Biomechanical Evaluation of Preoperative Rehabilitation in Patients of Anterior Cruciate Ligament (ACL) Injury”^1^, which we read with great interest. We congratulate the authors for their efforts. In this study, the authors investigated the biomechanical characteristics of patients with ACL injury by gait analysis, surface electromyography (SEMG), and proprioception test. They found that the deterioration of proprioception at 30° of the injured side will not recover and the non‐injured side will become worse after 1 year from the injury, indicating that the training for proprioception at 30° and vastus lateralis were important for rehabilitation, and the ACL reconstruction should be performed within 1 year. This finding is of value for clinicians in decision‐making. However, there are several concerns that need to be addressed.

First, the study mainly focused on comparison of biomechanical characteristics between the injured side and the non‐injured side at different injury stages. However, inter‐groups (i.e. Group A *vs* Group B) differences were not considered. To our knowledge, gait biomechanics after ACL injury change with time^2^.

Second, this study used the uninjured contralateral side as a control. However, it has been suggested in the literature that patients with unilateral ACL deficiency have altered kinematics of the contralateral knee^3^, ^4^, ^5^. Previously, a biomechanical test demonstrated that voluntary activation of the quadriceps femoris muscle on the ACL injured side as well as the contralateral quadriceps femoris muscle (Q‐ceps) was inhibited^6^. Therefore, the contralateral uninjured knee might not be a reliable normal kinematic control. We suggest that a control group should include healthy adult volunteers with no previous medical history of injury or surgery in the lower extremities, with match‐pairing for sex, age, height, and weight.

Third, we wonder if the authors might have insight as to the subgroup classification of ACL injured patients (copers and noncopers). Many studies have described kinematic and kinetic differences between copers (individuals who have the potential to compensate for the absence of an ACL without episodes of giving way after return to pre‐injury activities) and noncopers (those who have knee instability following ACL rupture with return to pre‐injury activities)^7^, ^8^, ^9^. Reduced Q‐ceps activity and altered walking patterns were not observed in copers^7^, ^8^. In contrast, significantly reduced Q‐ceps activity was found in non‐copers^9^. Therefore, the post‐injury activity level and knee stability should be evaluated to avoid selection bias.

In addition, it should be noted that there were some mistakes in the article. In the “Abstract” section, there is one extra word (“and”) in the first sentence of the “Conclusion” paragraph. In Table 2, the lines under “Group A” and “Group B” were marked incorrectly.

We appreciate that Li *et al*.^1^ have provided us with a clinically meaningful study. We would welcome comments by the authors as this would help to further support the findings of this important study.
